# TRIM55 inhibits colorectal cancer development via enhancing protein degradation of c‐Myc

**DOI:** 10.1002/cam4.6020

**Published:** 2023-05-22

**Authors:** Min Lin, Zejun Fang, Xuedan Lin, Weihua Zhou, Yizhang Wang, Shanshan Han, Ming Ye, Fengjiao Zhu

**Affiliations:** ^1^ Central Laboratory, Sanmen People's Hospital of Zhejiang Province Taizhou China; ^2^ Department of Pathology, Sanmen People's Hospital of Zhejiang Province Taizhou China; ^3^ Department of General Surgery, Sanmen People's Hospital of Zhejiang Taizhou China

**Keywords:** colorectal cancer, TRIM55, ubiquitination

## Abstract

**Background:**

Colorectal cancer (CRC) is one of the most common and lethal malignancies which including colon and rectum cancer. Tripartite motif containing 55 (TRIM55) is an E3 ubiquitin ligase belonging to the TRIM family. Although the aberrant TRIM55 expression has been implicated in several tumors, its functional role, and molecular mechanisms in CRC remain unknown.

**Methods:**

Immunohistochemical studies, qRT‐PCR, and Western blot were performed to analyze the expression of TRIM55 in CRC patients and cell lines. TRIM55 expression and its relevance to clinical traits and prognosis were further explored in the TCGA database, and in our 87 clinical samples. Subsequently, we performed a series of functional assays to explore the effect of TRIM55 on CRC progression. Finally, the molecular mechanism of TRIM55 was investigated by immunoprecipitation and ubiquitination analyses.

**Results:**

Here, we demonstrated that TRIM55 was markedly downregulated in CRC cell lines and tumors from CRC patients. Moreover, overexpression of TRIM55 could suppress CRC cell growth in vitro and inhibit CRC xenograft tumor development in vivo. Additionally, TRIM55 overexpression dampened CRC cell migration and invasion. Further bioinformatics analysis indicated that TRIM55 suppressed cyclin D1 and c‐Myc expression. Mechanistically, co‐immunoprecipitation assay revealed that TRIM55 directly interacted with c‐Myc and down‐regulated its protein expression level via protein ubiquitination. Intriguingly, c‐Myc overexpression partially antagonized the function of TRIM55 overexpression.

**Conclusions:**

Taken together, our findings suggest that TRIM55 inhibits CRC tumor development via, at least in part, enhancing protein degradation of c‐Myc. Targeting TRIM55 could provide a new therapeutic approach for CRC patients.

## Graphical abstract

Taken together, our findings suggest that TRIM55 inhibits CRC tumor development via enhancing protein degradation of c‐Myc. Targeting TRIM55 could provide a new therapeutic approach for CRC patients

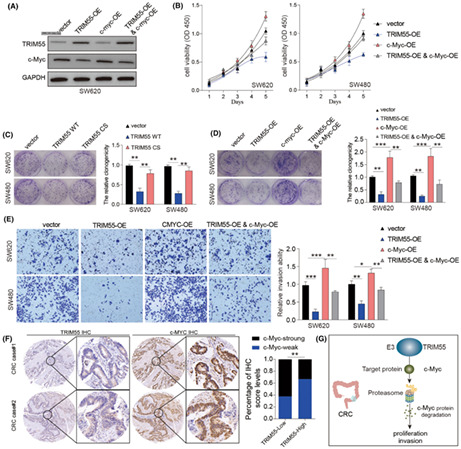

## 1 INTRODUCTION

Colorectal cancer (CRC), which includes both colon and rectum cancer, ranks the third place among the most common and lethal cancers.^1^ During the past decades, the treatment for CRC has been greatly advanced including surgical resection, radiotherapy, chemotherapy, and targeted therapy.^2^, ^3^ However, about a quarter CRC patients were diagnosed at tumor metastatic late stage and 5‐year survival remains very poor in patients with metastatic CRC despite the emerging of new therapies.^4^ Therefore, it is of great interest to better understand CRC development and metastasis to develop efficient therapeutic treatment for CRC patients.

Ubiquitination is a protein posttranslational process that involves in diverse physiological and pathological processes, especially tumorigenesis. Tripartite motif containing 55 (TRIM55) is an E3 ubiquitin ligase belonging to the TRIM family and is involved in the degradation of a series of crucial proteins.^5^, ^6^ The function of TRIM55 has been investigated in the development of cardiovascular system and muscle.^7^, ^8^, ^9^ Recent reports have established an anti‐tumor effect for TRIM55 in cancers. For instance, TRIM55 was found to suppress hepatocellular carcinoma tumorigenesis via regulating EMT and matrix metallopreoteinase‐2.^10^ A recent study revealed that TRIM55 enhanced protein degradation of Snail1 and inhibited lung adenocarcinoma development and metastasis.^11^ Nevertheless, the expression profile and underlying function of TRIM55 in CRC remains unidentified.

Here, we confirmed TRIM55 was markedly downregulated in CRC cells and tumors. Clinically, survival analysis indicated that low TRIM55 level was tightly associated with inferior prognosis. Functionally, overexpression of TRIM55 markedly slowed down the cell growth both in vitro and in vivo, and attenuated invasion/migration capabilities of CRC cells. In addition, we showed that TRIM55 directly interacted with c‐Myc and eventually deceased its expression via protein ubiquitination. More importantly, c‐Myc overexpression antagonized the function of TRIM55 and the inverse correlation between c‐Myc and TRIM55 levels was observed in clinical CRC cancer samples. Thus, our study suggests TRIM55 could be utilized to develop novel therapeutic strategy for CRC patients.

## 2 MATERIALS AND METHODS

### 2.1 Cell lines and treatment

Human CRC cells (DLD1, HCT116, SW620 and SW480) and HEK‐293 T cells were from the Cell Bank of CAS. Fetal human cells (FHC) from normal fetal colonic mucosa were from ATCC and used as control cell line. Cells were cultured in RPMI1640 supplemented with 10% FBS, antibiotics in an incubator (5% CO_2_, 37°C). Cycloheximide (CHX, 100 μg/mL, Sigma‐Aldrich) was added to cell culture medium to block protein synthesis, while MG132 (10 μM, Sigma‐Aldrich) was used to suppress proteasome degradation as indicated.

### 2.2 Patient specimen

Patients with newly diagnosed CRC were enrolled in this study between 2015 and 2019 year at Sanmen People's Hospital of Zhejiang Province. Fourteen CRC samples paired with adjacent normal tissues were collected and snap‐frozen in liquid N_2_ for subsequent analysis. Eighty‐seven CRC specimens and 22 paired adjacent control tissues were collected with formalin‐fixed for tissues microarray construction. Informed consent was provided by each patient. The Ethics Committee of Sanmen People's Hospital of Zhejiang Province reviewed and approved the study.

### 2.3 Transfection

Transfection was done using lipo 3000 following the manufacturer's manual. The si‐TRIM55 (si‐TRIM55#1: GCTACTTCTCAGGAGTTAGTA; si‐TRIM55#2: GCTTTGTGAGAAGTTTGATTA) and scramble control siRNA were obtained from Genecopoeia. The WT TRIM55 and mutated TRIM55 CS cDNAs were cloned and constructed into pcDNA3.1 vector. Overexpression vector of c‐Myc, His‐tagged c‐Myc and HA‐tagged TRIM55 were obtained from GeneWiz.

### 2.4 Immunofluorescence staining

SW620 and SW480 were fixed in paraformaldehyde and permeabilization with triton X‐100 after different treatments. Then, cells were incubated with 1% BSA for 1 h for blocking, and subsequently incubation with TRIM55 and c‐Myc antibodies overnight. After DAPI (Sigma) staining, the slides were analyzed for immunofluorescence intensity using a confocal microscope.

### 2.5 Immunohistochemical (IHC) staining

The immunohistochemical staining was conducted as previously stated.^12^ Briefly, tumor tissues were cut into 5 μm slides and followed by deparaffinization and rehydration. Slices were blocked with BSA, stained with primary antibodies against KI67 (F802546, OriGene) and PCNA (800894AM, OriGene), and subsequently visualized with HRP conjugated secondary antibodies using a DAB kit.

### 2.6 Western blot

SDS‐PAGE and western blot were conducted using the following antibodies: anti‐GAPDH (BM3874, Boster), anti‐TRIM55 (A15917, Abclonal), anti‐MMP2 (orb12416, Biorbyt), anti‐MMP9 (27306‐1‐AP, Proteintech), anti‐N‐Cadherin (22018‐1‐AP, Proteintech), anti‐Bak (A0498, Abclonal), anti‐Bax (sc‐7480), anti‐Bcl2 (ET7110‐51, HuaBio), anti‐Caspase 3 (ET1602‐39, HuaBio), anti‐c‐Myc (AF0358, Affinity), anti‐p21/Cip1 (27296‐1‐AP, Proteintech), anti‐E‐Cadherin (20874‐1‐AP, Proteintech), anti‐P27/Kip1 (NBP1‐32213, Novus), anti‐survivin (NBP2‐48494, Novus), mouse anti‐HA‐Tag mAb (AE065, Abclonal), and mouse anti His‐Tag mAb (AE003, Abclonal). Protein bands were developed with a chemiluminescence kit (ThermoFisher).

### 2.7 RT‐qPCR

RNA was extracted and equal amount of RNA was reverse‐transcribed to generate complementary DNA. Quantitative PCR was conducted to quantify the mRNA expression by using SYBR1 Premix Ex Taq™ II (Takara). Primers used in the study were as following: GAPDH: GGAGCGAGATCCCTCCAAAAT and GGCTGTTGTCATACTTCTCATGG; TRIM55: TGGTTTTGGATAGACATGGGGT and CTGGTGGACTCCTGCTTGTA; c‐Myc: TCCCTCCACTCGGAAGGAC and CTGGTGCATTTTCGGTTGTTG.

### 2.8 Cell growth assays

Cell growth was analyzed by CCK‐8, colony formation and 5‐ethynyl‐20‐deoxyuridine (EDU) DNA incorporation assay, respectively.^13^, ^14^

### 2.9 Cell migration and invasion assay

Cell invasion and migration capability were evaluated by transwell and wound‐healing experiments.^13^, ^14^

### 2.10 CRC xenograft tumor model

Briefly, 12 BALB/c female nude mice (6–8 weeks) were purchased from Vital River Laboratory. 5 × 10^6^ CRC cells were inoculated into mice subcutaneously. Tumor growth was measured every 4 days and tumor volume was calculated by using formula *L* (length) × *W* (width) × *W* (width) × 1/2. Animal studies were approved by the Experimental Animal Ethics Committee of the Sanmen People's Hospital of Zhejiang Province.

### 2.11 Hematoxylin & eosin (H&E) staining

Xenograft SW620 tumor were formalin‐fixed, paraffin‐embedded, and sectioned into 6 μm slides. Then slides were stained with H&E and tumor structure/cell infiltration were analyzed under a microscope.

### 2.12 Co‐immunoprecipitation assay

Cell lysates of SW620 and SW480 were incubated with anti‐TRIM55 (A15917, Abclonal) at 4°C overnight, following with pull‐down assay using protein A/G beads. The immunoprecipitated c‐Myc and TRIM55 proteins were examined by using western blot.

### 2.13 Ubiquitination assay

SW620 cells were treated with MG132 for 6 h to block the protein degradation. Cell lysates were prepared and ubiquitinated c‐Myc was purified by immunoprecipitation for further analysis using immunoblot.

### 2.14 Statistical analysis

In the statistical analysis for the experiments, all data were shown in the form of mean ± SD (standard deviation, three independent experiments), and analyzed using GraphPad (Prism). Student's *t*‐test and one‐way ANOVA were used respectively for significance analysis of two‐group and more‐than‐two‐group data. Survival analysis was performed by Kaplan–Meier curves and log‐rank test for significance. *p* < 0.05 was considered to be statistically significant of the difference.

## 3 RESULTS

### 3.1 TRIM55 expression is downregulated in CRC tissues

**FIGURE 1:**
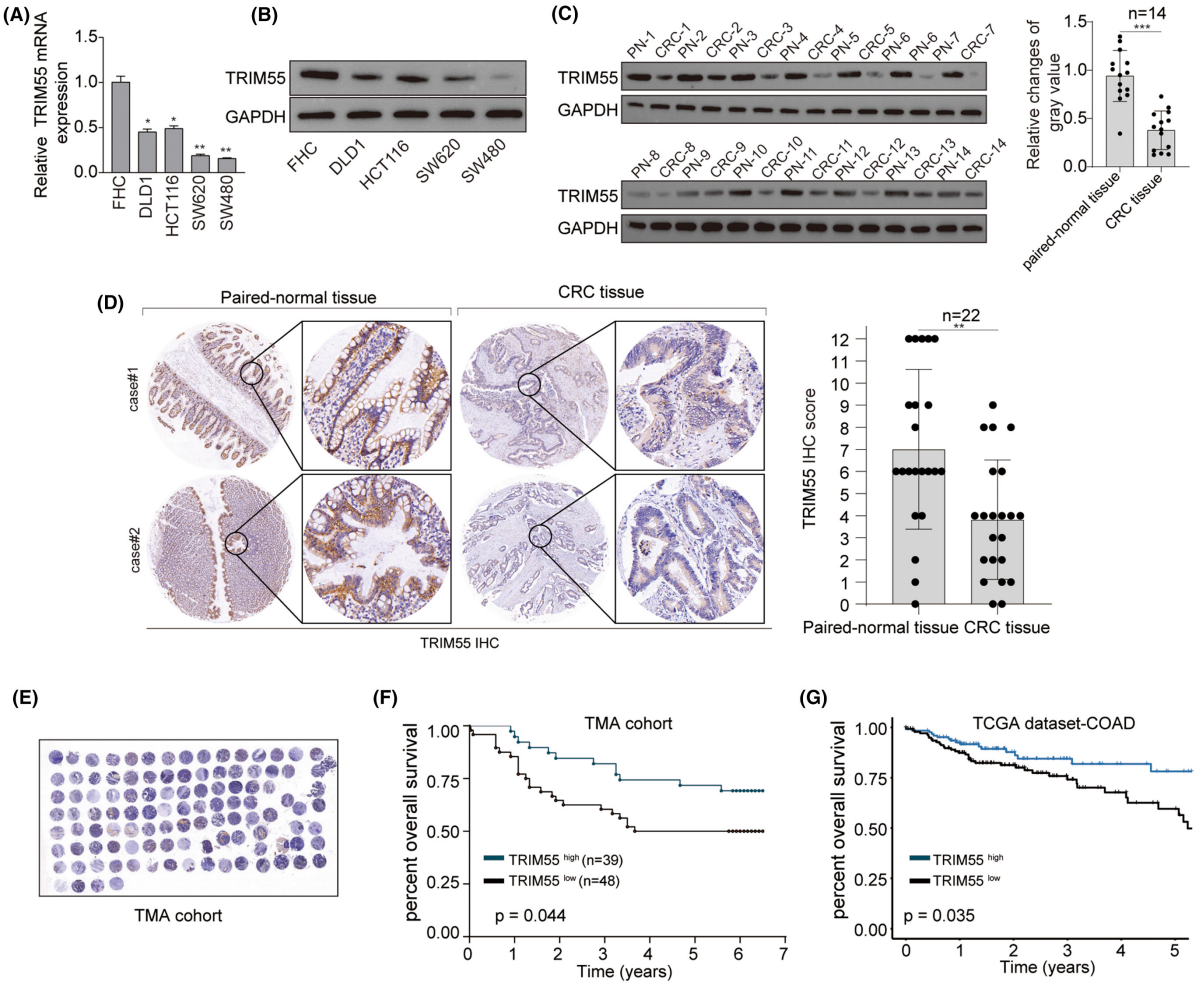
TRIM55 expression is downregulated in CRC. (A,B) The mRNA (A) and (B) protein levels of TRIM55 were analyzed in CRC cell lines (DLD1, HCT116, SW620, and SW480) and fetal colon cell line FHC. (C) The protein expression of TRIM55 was analyzed in CRC tissues and paired‐normal tissues from 14 CRC patients. (D) IHC staining of TRIM55 was conducted in CRC tissues and paired‐normal tissues from 22 CRC patients. (E,F) Tissue microarray (TMA) was performed and the prognosis of patients with high or low expression of TRIM55 was analysis in CRC TMA cohort. (G) Kaplan–Meier analysis was performed using TCGA dataset colon adenocarcinoma (COAD) cohort based on TRIM55 expression levels. *p < 0.05, **p < 0.01, ***p < 0.001.

TRIM55 expression was examined in CRC cell lines and fetal colon cell line FHC. As demonstrated by RT‐qPCR and Western blot, TRIM55 were significantly downregulated in CRC cell lines (DLD1, HCT116, SW620, and SW480) compared with that in FHC cells (Figure 1A,B). In support of above findings, both western blot and IHC staining revealed that TRIM55 expression was markedly downregulated in CRC tissues (Figure 1C,D). We performed tissue microarray to address the clinical significance of TRIM55 expression in CRC patients and the results indicated low expression of TRIM55 was strongly linked with worse postsurgical outcome of CRC patients (Figure 1E,F). Similarly, Kaplan–Meier analysis using TCGA dataset‐COAD also demonstrated that high TRIM55 expression suggested a favorable prognosis in CRC patients (Figure 1G).

### 3.2 Overexpression of TRIM55 suppresses CRC cell growth

**FIGURE 2:**
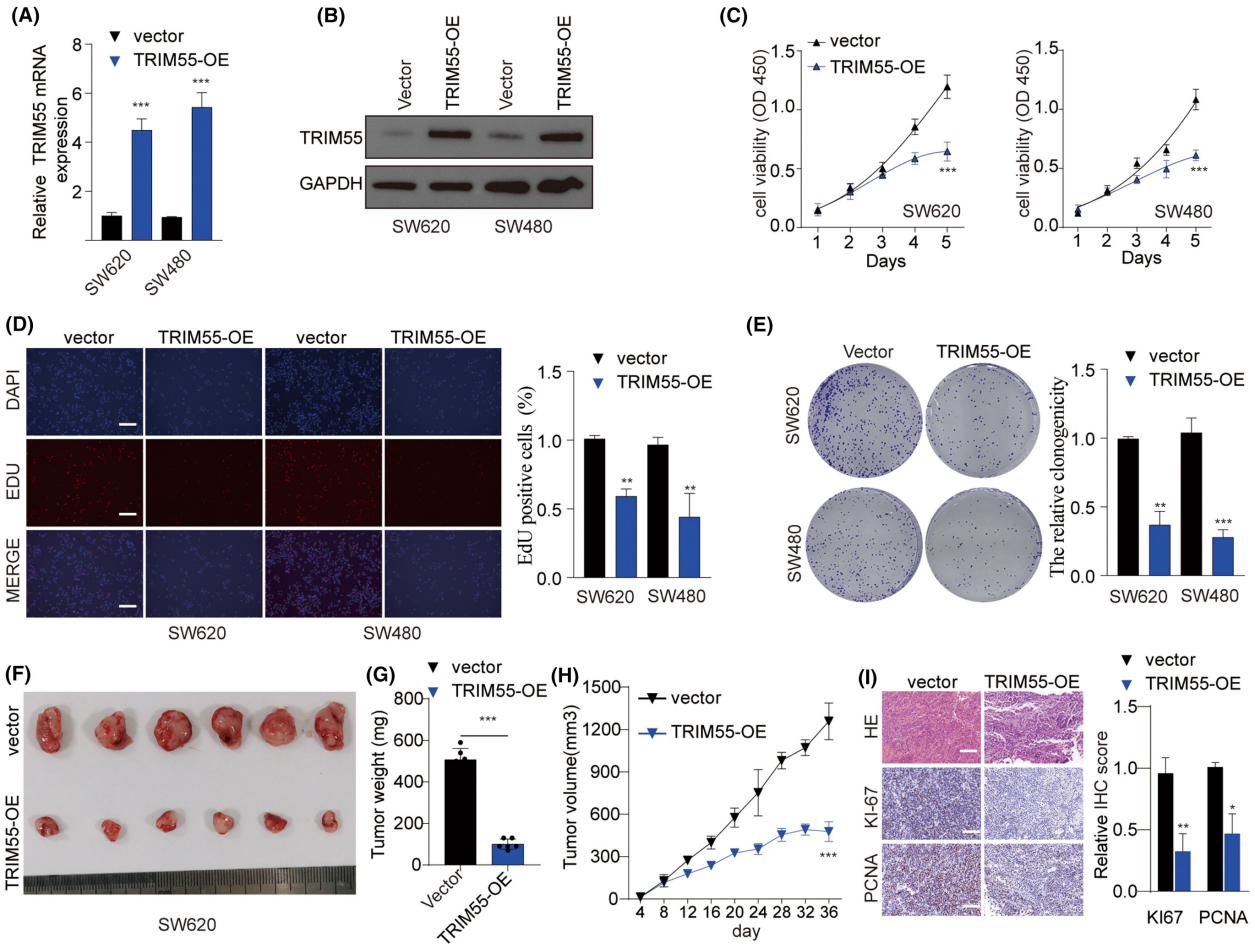
Overexpression of TRIM55 suppresses CRC cell growth. (A–E) SW620 and SW480 cells were transfected with empty vector or TRIM55 overexpression vector (TRIM55‐OE). The relative mRNA (A) and protein (B) levels of TRIM55 were examined by qPCR and western blot 48 hlater. (C) Cell proliferation, (D) DNA incorporation, and (E) colony formation of SW620 and SW480 cells were analyzed by CCK‐8 assay, EDU DNA incorporation assay, and colony formation assay respectively. (F–I) SW620 cells were transfected with empty vector or TRIM55‐OE. 5 × 10^6^ cells were inoculated into nude mice to establish the CRC xenograft tumor model. After 36 days, tumors were harvested (F) and weighted (G). Tumor growth was measured every 4 days and the growth curve was plotted (H). (I) H&E and IHC staining of KI‐67 and PCNA were performed to evaluate the cell proliferation in tumor. *p < 0.05, **p < 0.01, ***p < 0.001.

To explore the function of TRIM55, we overexpressed TRIM55 in CRC cell line SW620 and SW480 (Figure 2A,B). Overexpression of TRIM55 inhibited SW620 and SW480 cell growth capacity and suppressed colony formation of SW620 and SW480 cells (Figure 2C–E). We established CRC xenograft tumor model using SW620 cell transfected with empty vector or TRIM55 overexpression vector. In parallel, TRIM55 overexpression dramatically suppressed tumor growth (Figure 2F–H). Consistently, IHC staining of KI‐67 and PCNA indicated that ectopic TRIM55 expression inhibited SW620 cell proliferation in vivo (Figure 2I).

### 3.3 Overexpression of TRIM55 decelerates in vitro migration and invasion of CRC

**FIGURE 3:**
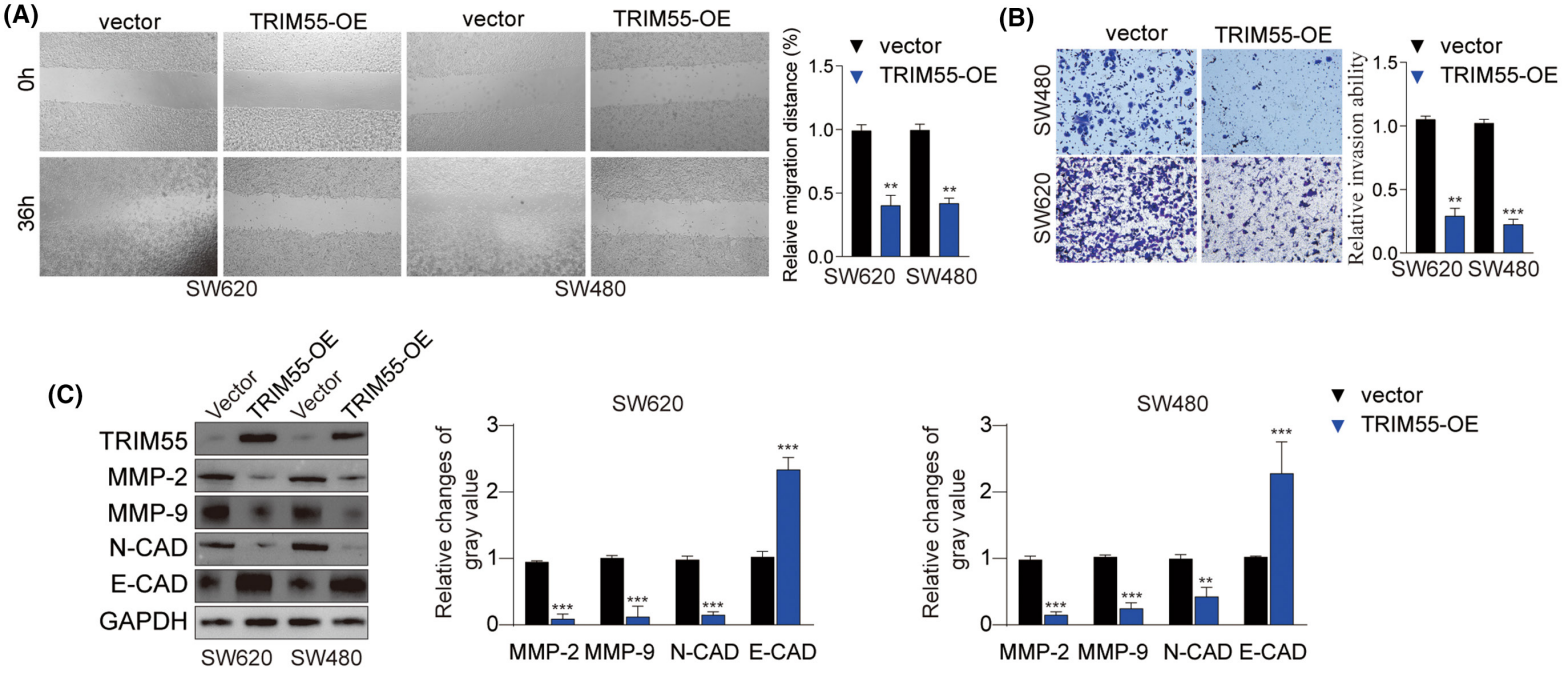
Overexpression of TRIM55 inhibits CRC cell migration and invasion. (A–C) SW620 and SW480 cells were transfected with empty vector or TRIM55‐OE. (A) Cell migration was analyzed by wound‐healing assay. (B) Cell invasion was analyzed by transwell assay. (C) The protein expression of TRIM55, MMP‐2, MMP‐9, E‐CAD, N‐CAD, and GAPDH were analyzed by western blot and quantified. *p < 0.05, **p < 0.01, ***p < 0.001.

We further investigated the impact of TRIM55 on metastatic potential of CRC cells. Wound‐healing assay revealed that upregulation of TRIM55 expression attenuated cell migration of SW480 and SW620 (Figure 3A). Transwell assay demonstrated that TRIM55 overexpression dampened cell invasion capability (Figure 3B). In consistent, ectopic TRIM55 expression noticeably suppressed the expression of invasion‐related proteins (MMP‐2, MMP‐9, N‐CAD), while enhanced the expression of E‐CAD (Figure 3C). The aforementioned findings strongly implicated that TRIM55 overexpression hampered the invasion of CRC cells.

### 3.4 TRIM55 overexpression suppresses cyclin D1 and c‐Myc

**FIGURE 4:**
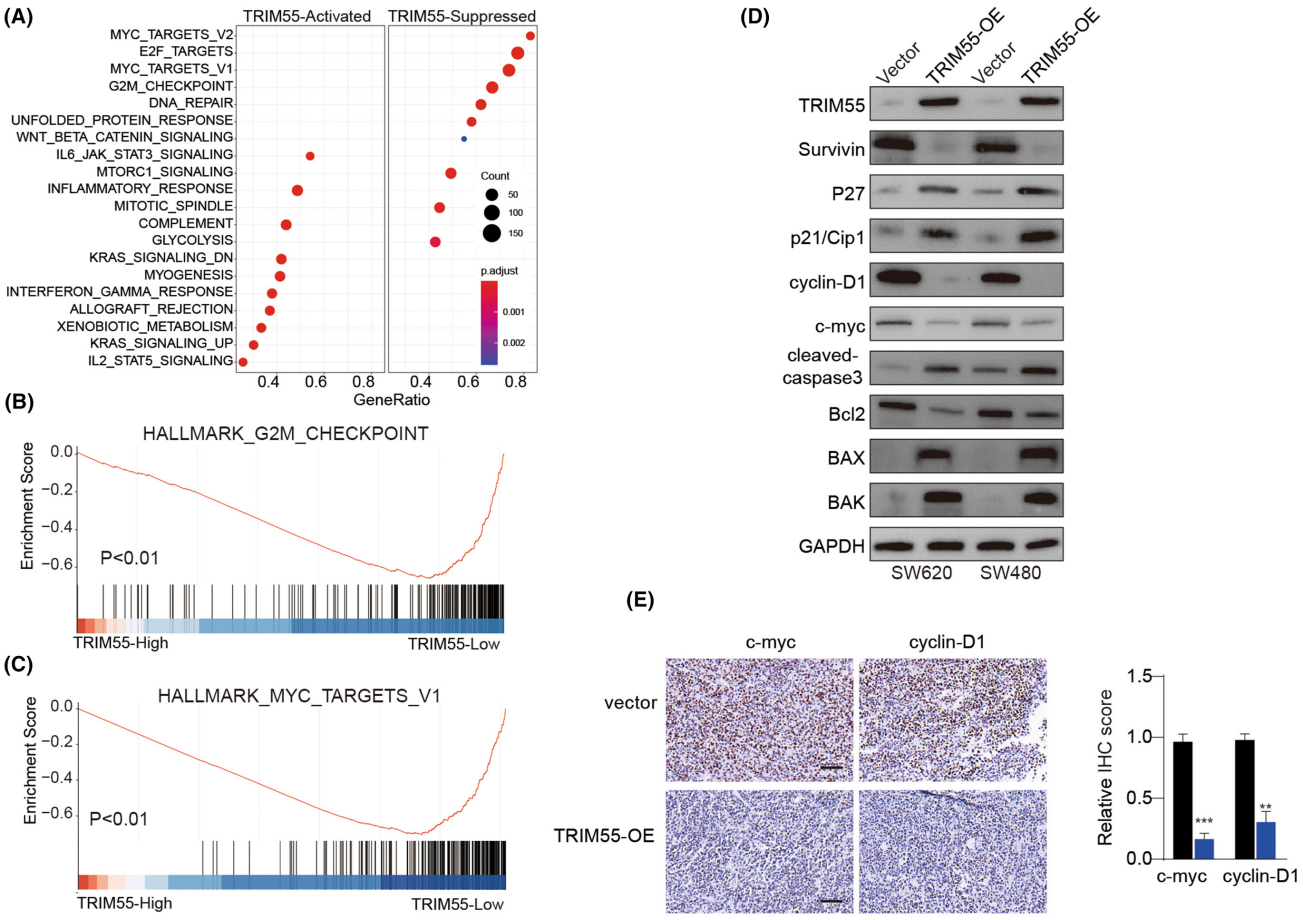
TRIM55 overexpression suppresses cyclin D1 and c‐Myc. (A) Gene Ontology (GO) enrichment analysis of the top 800 genes with TRIM55‐activated or TRIM55‐Suppressed correlation coefficient. (B,C) Gene set enrichment analysis (GSEA) of the enriched signaling pathways with TRIM55‐High or TRIM‐55‐low expression using Hallmark signaling dataset. (D) SW620 and SW480 cells were transfected with empty vector or TRIM55‐OE. The protein expression levels of TRIM55, survivin, P27, p21/Cip1, Cyclin D1, c‐Myc, cleaved‐caspase 3, Bcl2, BAX, BAK, and GAPDH were examined by western blot. (E) IHC staining of c‐Myc and cyclin D1 was performed using SW620 xenograft tumor tissues. *p < 0.05, **p < 0.01, ***p < 0.001.

To investigate how TRIM55 regulates CRC development and invasion, gene ontology analysis and gene set enrichment analysis were employed. As illustrated in Figure 4A–C, high expression TRIM55 was associated with enrichment of genes involved in cell cycle G2M checkpoint and Myc signaling. In addition, we found that overexpression of TRIM55 enhanced the expression of p21/Cip1, P27, cleaved‐caspase3, BAX, and BAK while suppressed the expression of Bcl2, indicating the significant role of TRIM55 in regulating cell cycle and cell apoptosis (Figure 4D). Moreover, TRIM55 markedly weakened survivin, cyclin D1, and c‐Myc expression (Figure 4D). Similarly, IHC staining of c‐Myc and cyclin D1 using SW620 xenograft tumor tissues revealed that overexpression of TRIM55 inhibited c‐Myc and cyclin D1 expression levels in tumors (Figure 4E).

### 3.5 TRIM55 directly interacts with c‐Myc

**FIGURE 5:**
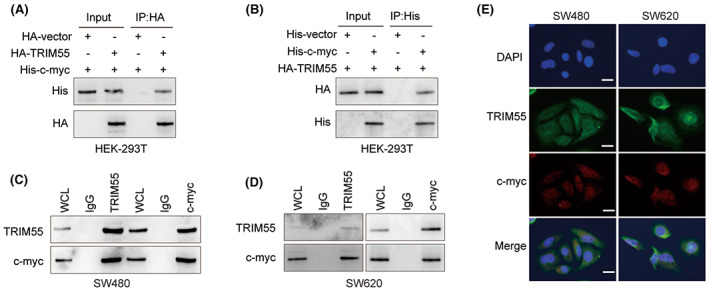
TRIM55 directly interacts with c‐Myc. (A,B) HEK‐293 T cells were transfected with HA‐vector, HA‐TRIM55, or His‐c‐Myc. Co‐IP assay was done using anti‐HA (A) or anti‐His (B) antibody. The immunoprecipitated protein was analyzed by western blot. (C,D) Co‐IP assay was performed in SW620 or SW480 cell lysate using anti‐TRIM55 or anti‐c‐Myc antibody. The immunoprecipitated protein was analyzed by western blot. (E) Immunofluorescence staining of c‐Myc and TRIM55 was performed in SW620 and SW480 cells. DAPI staining was used to localize the nucleus.

To further investigate the functional relationship between TRIM55 and c‐Myc, Co‐IP assay was conducted in HEK‐293 T cells overexpressed with HA‐TRIM55 and His‐c‐Myc. The results showed that TRIM55 was co‐immunoprecipitated with c‐Myc (Figure 5A,B). In addition, Co‐IP assay in SW620 and SW480 cells demonstrated that TRIM55 directly interacted with endogenous c‐Myc (Figure 5C,D). Moreover, immunofluorescence staining revealed that TRIM55 was co‐localized with c‐Myc in SW620 and SW480 cells (Figure 5E).

### 3.6 TRIM55 decreases the expression level of c‐Myc via protein ubiquitination

**FIGURE 6:**
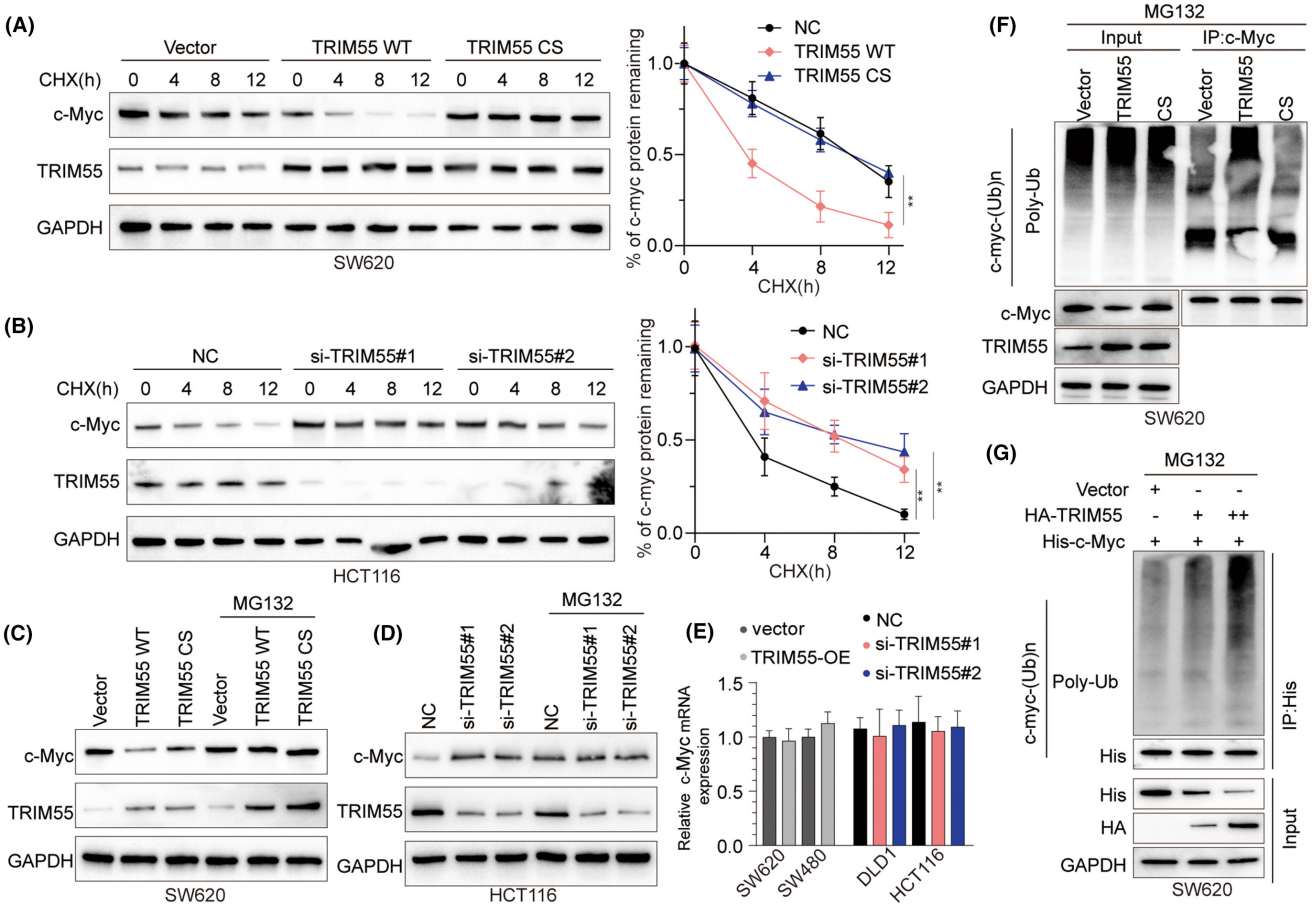
TRIM55 decreases the expression level of c‐Myc via protein ubiquitination. (A) SW620 transfected with empty vector, TRIM55‐WT, or TRIM55‐CS was treated with cycloheximide (CHX). c‐Myc, TRIM55, and GAPDH levels in SW620 cells were analyzed via western blot. (B) HCT116 cells transfected with si‐NC, si‐TRIM55#1, or si‐TRIM55#2 were treated with CHX for indicated times. c‐Myc, TRIM55, and GAPDH levels in SW620 cells were analyzed via western blot. (C) SW620 cells transfected with empty vector, TRIM55‐WT, TRIM55‐CS, were treated with or without MG132. c‐Myc, TRIM55, and GAPDH levels in SW620 cells were analyzed via western blot. (D) HCT116 cells transfected with si‐NC, si‐TRIM55#1, si‐TRIM55#2, were treated with or without MG132. c‐Myc, TRIM55, and GAPDH in HCT116 cells were analyzed via western blot. (E) SW620 or SW480 cells were transfected with empty vector or TRIM55 overexpression vector. DLD1 or HCT116 cells were transfected with si‐NC, si‐TRIM55#1, or si‐TRIM55#2. The c‐Myc mRNA levels were analyzed by qPCR 48 h later. (F) SW620 cells were transfected with control vector, TRIM55 WT, or TRIM55 CS. After 48 h, cell lysates were performed co‐IP using anti‐c‐Myc antibody. The precipitated proteins were probed with Poly‐Ub, c‐Myc, TRIM55, and GAPDH. (G) SW620 cells were transfected with empty vector, His‐c‐Myc, with or without HA‐TRIM55. After 48 h, cell lysates were performed co‐IP using anti‐His antibody. The precipitated proteins were probed with Poly‐Ub, His, HA, and GAPDH.

To understand how TRIM55 regulates c‐Myc, SW620 cells were transfected with WT TRIM55 overexpression vector, or TRIM55 with CS mutation vector and negative control. Cells were treated with CHX to block the protein biosynthesis. As shown in Figure 6A, overexpression of TRIM55 significantly enhanced c‐Myc downregulation while overexpression TRIM55 CS abolished c‐Myc reduction. In contrast, knockdown of TRIM55 in HCT116 cells suppressed c‐Myc downregulation (Figure 6B). We also used MG132 to block the proteasome degradation, which led to sustained levels of c‐Myc in SW620 cells (Figure 6C). In addition, MG132 abolished the upregulation of c‐Myc in HCT116 cells transfected with si‐TRIM55 (Figure 6D). Overexpression or knockdown of TRIM55 did not affect the mRNA levels of c‐Myc in CRC cell lines, indicating the regulation of c‐Myc was at translational/posttranslational level (Figure 6E). Co‐IP using c‐Myc antibody revealed that overexpression of TRIM55 enhanced c‐Myc ubiquitination while overexpression of TRIM55 CS had no effect on c‐Myc ubiquitination (Figure 6F). Similarly, we overexpressed HA‐TRIM55 and His‐c‐Myc in SW620 cells. Co‐IP using His antibody demonstrated that overexpression of TRIM55 decreased c‐Myc levels via promoting protein ubiquitination (Figure 6G).

### 3.7 Overexpression of c‐Myc partially antagonizes the function of TRIM55 overexpression

**FIGURE 7:**
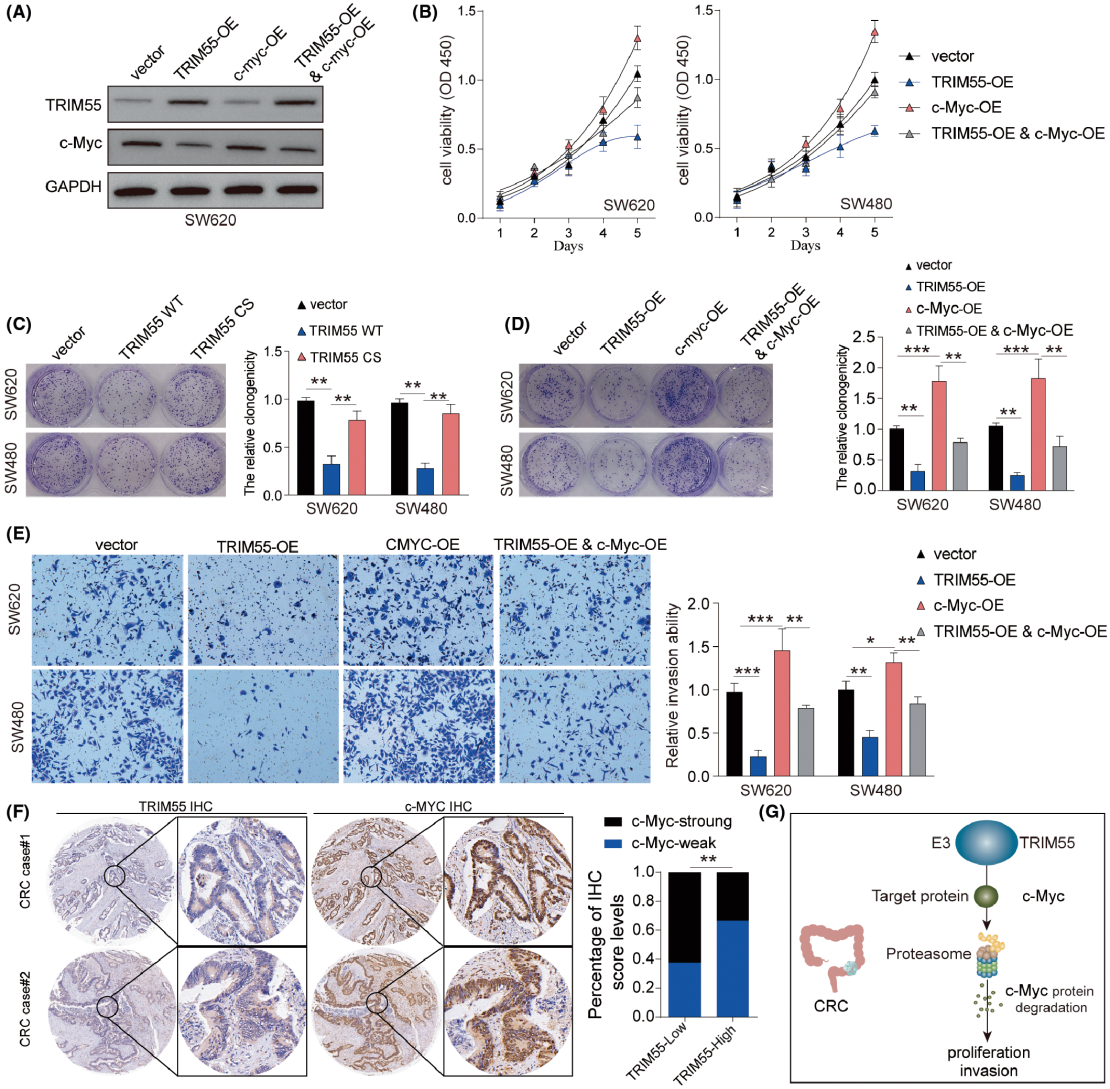
Overexpression of c‐Myc partially antagonizes the function of TRIM55 overexpression. (A–E) SW620 or SW480 cells were transfected with vector, TRIM55 overexpressing vector, c‐Myc overexpressing vector, or both TRIM55 and c‐Myc overexpressing vector. (A) The expression levels of TRIM55, c‐Myc and GAPDH were analyzed by western blot 48 h later. (B) Cell viability was assessed by CCK‐8 kit. (D) Cell growth was assessed by colony formation assay. (E) Cell invasion was analyzed by transwell assay. (C) SW620 and S480 cells were transfected with control vector, TRIM55 WT or TRIM55 CS overexpressing vectors. Cell growth was evaluated by colony formation assay. (F) IHC staining of TRIM55 and c‐Myc was conducted using CRC tissues and the IHC score of c‐Myc in TRIM55‐low or TRIM55‐high tissues were analyzed. (G) The graph depicted the potential regulation of c‐Myc degradation by TRIM55 in CRC. *p < 0.05, **p < 0.01, ***p < 0.001.

To verify whether overexpression c‐Myc could reverse the function of TRIM55 overexpression, we overexpressed TRIM55, c‐Myc, or TRIM55 and c‐Myc in SW620 cells (Figure 7A). While overexpression of TRIM55 markedly suppressed cell growth and invasion of SW620 and SW480 cells, c‐Myc overexpression enhanced CRC cell growth and invasion capability and antagonized the function of TRIM55 (Figure 7B, D,E). Intriguingly, overexpression of TRIM55 CS did not alter the colony formation of CRC cells (Figure 7C). IHC staining of TRIM55 and c‐Myc using CRC tumor tissues demonstrated that TRIM55 low was associated with strong c‐Myc staining (Figure 7F). Thus, we found that TRIM55 regulated CRC development via enhancing c‐Myc degradation.

## 4 DISCUSSION

After decades of development, great advances have been made with therapeutic treatment for CRC patient.^3^ Better understanding of CRC tumorigenesis and metastasis could lead to identify novel target and develop innovative therapies.^15^, ^16^ In the present study, we demonstrated that TRIM55 plays a tumor suppressor role in CRC. Both CRC cell line and patient tumors showed down‐regulation of TRIM55 expression while low levels of TRIM55 predicted worse prognosis in patients with CRC. Gain‐of‐function in vitro and in vivo experiments proved that TRIM55 overexpression inhibited the malignancy phenotype of CRC cells and dampened CRC xenograft tumor development. Further analysis revealed that TRIM55 was involved with cell cycle arrest and Myc signaling pathway. It has been well documented that c‐Myc is one of the most important oncoproteins and could promote tumorigenesis in multiple cancers including CRC.^17^ Intriguingly, mechanistic study revealed that TRIM55 obviously triggered the protein ubiquitination of c‐Myc. Taken together, targeting TRIM55/c‐Myc could be utilized to develop new therapies for CRC patients.

TRIM55 is one of the TRIM family proteins as an E3 ubiquitin ligase, which exerts its function via ubiquitination.^6^ TRIM55 was reported to prevent diabetic cardiomyopathy via posttranslational ubiquitination of PPAR expression.^9^ Another study showed that TRIM55 degraded DUSP1 and enhanced cell apoptosis in cardiac I/R injury.^18^ It is worthy to note that emerging evidence has revealed the TRIM55 functional as tumor suppressor in hepatocellular carcinoma and lung adenocarcinoma through targeting matrix metalloproteinase‐2 (MMP2) or Snail1 in a E3 ubiquitin ligase activity‐dependent manner, respectively.^10^, ^11^ Consistent with previous findings, our findings suggest that TRIM55 inhibits CRC development and invasion via ubiquitination of c‐Myc protein. c‐Myc was reported to be an oncogene in CRC.^12^, ^19^, ^20^ In CRC stem cells, c‐Myc could maintain the self‐renewal and chemo‐resistance character which could be an effective target for CRC treatment.^13^

Indeed, it has been extensively reported that ubiquitination process contributes to the aberrant degradation of c‐Myc in tumors. Targeting the MYC ubiquitination could provide a unique therapeutic strategy for cancer therapy.^14^ For instance, eleven‐nineteen lysine‐rich leukemia (ELL) was reported to function as an E3 ubiquitin ligase targeting c‐Myc for proteasomal degradation.^21^ In line with the above findings, we revealed that E3 ligase TRIM55 has a direct interaction with c‐Myc identified by Co‐IP assays and immunofluorescence staining assays, while mediated the polyubiquitination of c‐Myc and its subsequent degradation in the proteasome in CRC cells. In detail, TRIM55 degrades c‐Myc via ubiquitination to exert tumor‐suppressive functions in CRC progression. Given c‐Myc's potential involvement in cancer, multiple strategies have been employed to tackle the c‐Myc oncogene such as Myc/Max disruption, MYC transcription inhibition and protein destabilization.^22^, ^23^ All the above findings further corroborated the notion that TRIM55 activation may be an effective means of targeting c‐Myc‐dependent tumors, a goal that has been particularly elusive with other approaches.

There are a few limitations in the study. First, though we validated the function of TRIM55 via different approaches, more studies are needed to further confirm the tumor suppressor function of TRIM55 such as using TRIM55 gene knockout mice. Second, besides the c‐Myc protein, TRIM55 also regulates multiple factors involving CRC development and invasion, which requires further investigation. Moreover, c‐Myc function has not been extensively studied in the CRC models.

## 5 CONCLUSION

In summary, our findings indicate that TRIM55 is a critical tumor suppressor and exerts its anti‐tumor function via ubiquitination of the oncogene c‐Myc in CRC. This novel mechanism may provide novel approaches to explore treatment for CRC through restoring TRIM55 expression in CRC cases with c‐Myc activation. Further studies explore the potential therapeutic strategies targeting TRIM55/c‐Myc should be implemented.

## AUTHOR CONTRIBUTIONS

**Min Lin:** Data curation (equal); investigation (equal); writing – original draft (equal); writing – review and editing (equal). **Zejun Fang:** Resources (equal); software (equal). **Xuedan Lin:** Data curation (equal); formal analysis (equal). **Weihua Zhou:** Investigation (equal); methodology (equal). **Yizhang Wang:** Validation (equal); visualization (equal). **Shanshan Han:** Funding acquisition (equal); investigation (equal). **Ming Ye:** Writing – original draft (equal); writing – review and editing (equal). **Fengjiao Zhu:** Supervision (equal); writing – original draft (equal).

## FUNDING INFORMATION

This study was supported by grants: Zhejiang Provincial Natural Science Foundation of China (LQ20H160004, LQ21H160009); Taizhou Science and Technology Plan (1902KY192, 20ywb183, 21ywb156, 22ywb170); Zhejiang Medical and Health Science and Technology Plan (2023KY1352); Science and Technology Program of Sanmen County Public Technology Social Development Project (18304). The funding body had no role in the present study.

## CONFLICT OF INTEREST STATEMENT

No conflicts of interest were declared by the authors.

## ETHICS STATEMENT

The study was approved by the ethical committee of Sanmen People's Hospital of Zhejiang Province.

## Data Availability

Data sharing is not applicable to this article as no new data were created or analyzed in this study.
